# Supplementary material for: c-Myc uses Cul4b to preserve genome integrity and promote antiviral CD8+ T cell immunity
Source: Nat Commun. 2023 Nov 4;14:7098. doi: 10.1038/s41467-023-42765-7 (PMC10625626; doi:10.1038/s41467-023-42765-7)
Supplement: Supplementary file 1 — Supplementary information [file 41467_2023_42765_MOESM1_ESM.pdf]

Supplementary Information

c-Myc uses Cul4b to preserve genome integrity and promote antiviral CD8+ T cell immunity

Asif A. Dar, Dale Kim, Scott Gordon, Kathleen Klinzing, Siera Rosen, Ipsita Guha, Nadia Porter, Yohaniz Ortega, Katherine Forsyth, Jennifer Roof, Hossein Fazelinia, Lynn A. Spruce, Laurence C. Eisenlohr, Edward Behrens, Paula M. Oliver

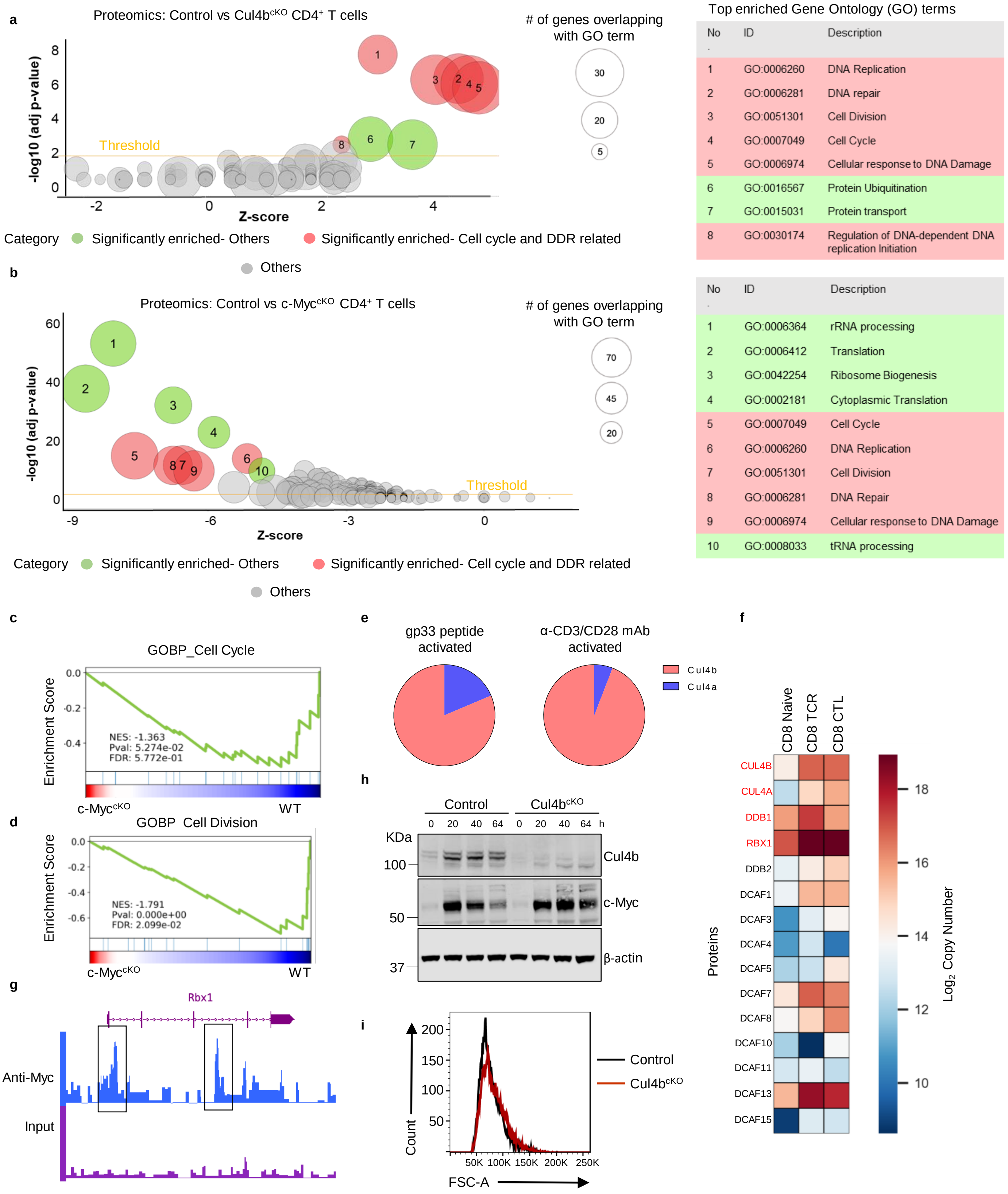

Supplementary Figure 1: c-Myc controls cell cycle and DDR pathways by remodeling T cell proteome. a, b) Quantitative proteomics analysis was done on the Cul4b deleted (Cul4b^{cKO}) versus the control CD4^{+} T cells restimulated for 4 h with anti-CD3/CD28 mAbs; And on c-Myc deleted (c-Myc^{cKO}) and control CD4^{+} T cells stimulated for 24 h with anti-CD3/CD28 mAbs. DAVID functional annotation tool was used to perform the gene-annotation enrichment analysis on the set of differentially expressed proteins (adjusted p-value < 0.05) between Cul4b^{cKO} or c-Myc^{cKO} vs control CD4^{+} T cells. The bubble plot depicts the enriched functional networks of proteins. The z-score is assigned to the x-axis and the negative logarithm of the adjusted p-value to the y-axis (the higher the more significant). The area of the displayed circles is proportional to the number of genes assigned to the term and the red color corresponds to the significantly enriched GO terms related to cell cycle and DNA damage response within the top ten terms. The green color in Cul4b deleted (Cul4b^{cKO}) and control CD4^{+} T dataset corresponds to protein ubiquitination and transport, while in c-Myc^{cKO} vs control CD4^{+} T dataset, it corresponds to Translation and Transcription related terms among top ten GO terms. Grey corresponds to the other GO terms. **c, d)** The gene set enrichment analysis (GSEA) identifies enrichment of GO biological processes related to Cell Cycle and Cell Division in control over c-Myc^{cKO} CD8^{+} T cells. **e)** The pie chart shows the relative proportion of Cul4a and Cul4b in antigen (gp33) activated and anti-CD3/CD28 mAb activated CD8^{+} T cells. The relative proportion is calculated from the mean copy numbers of Cul4a and Cul4b from analysis of the three different samples. **f)** Heatmap shows the protein abundance (copy number's) of CRL4B components in naïve, gp33^{+} activated CD8^{+} T cells and cytotoxic T lymphocytes (CTLs). **g)** c-Myc ChIP-seq tracks at the *Rbx1* gene locus in WT CD8^{+} T cells (top, gene structures and transcriptional orientations). c-Myc binding peaks, identified by the Model based Analysis for ChIP-seq (MACS) algorithm, are marked by rectangles. **h)** TCR driven activation of c-Myc and Cul4b. Naive CD8^{+} T cells from control and Cul4b^{cKO} mice were activated by anti-CD3/CD28 mAbs. At indicated time points after activation, cell lysates were prepared and expression of c-Myc and Cul4b was assessed by immunoblot. Data across the lanes was normalized to β-actin and data is representative of three independent experiments. **i)** Forward scatter area (FSC-A) of 48 h anti-CD3/CD28 mAb activated control and Cul4b^{cKO} CD8^{+} T cells. The data is representative of four independent experiments. Source data are provided as a "Source Data" file

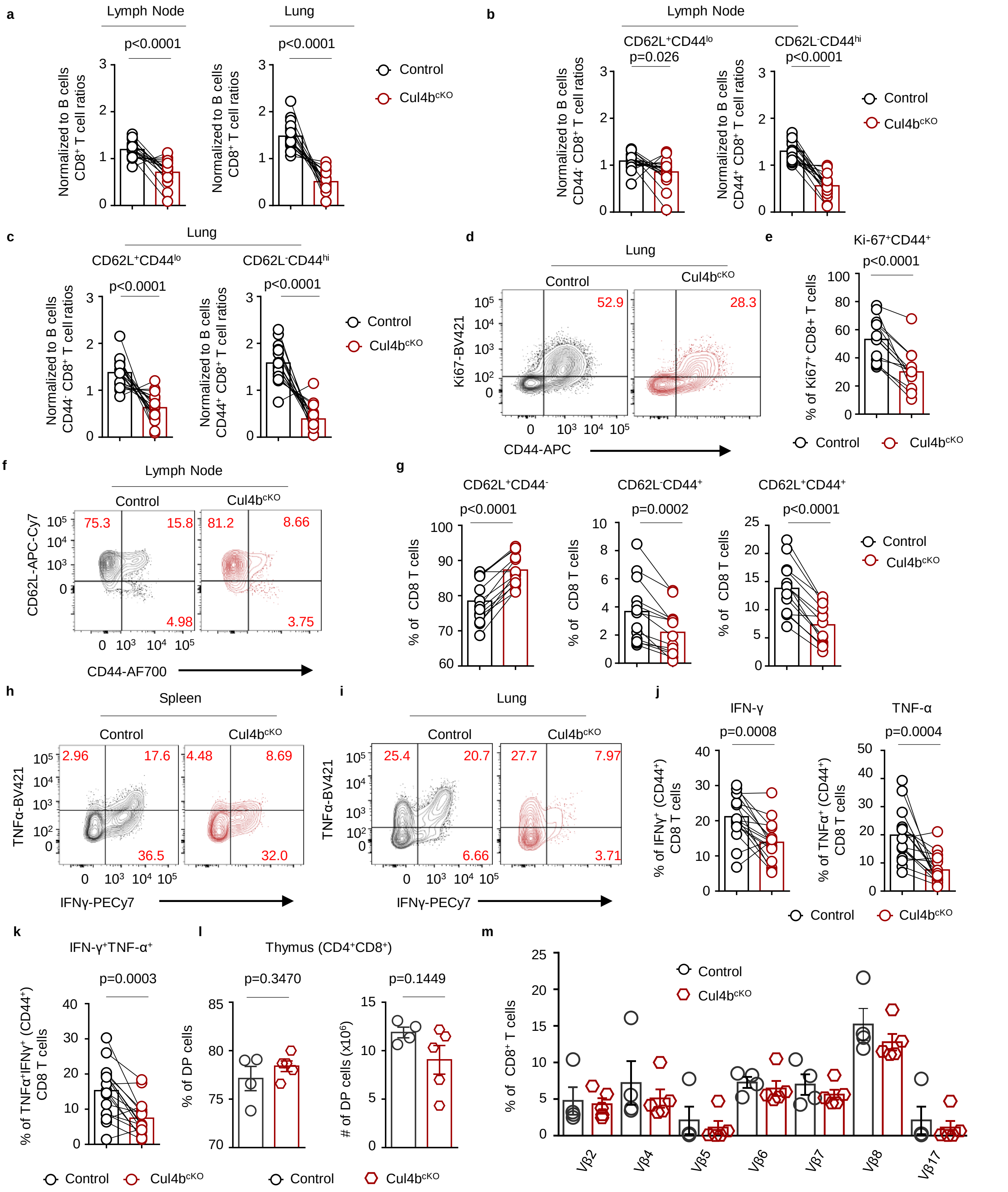

Supplementary Figure 2: Cul4b is required for the maintenance of activated and effector CD8$^+$ T cells: a) The data shows the comparison of the CD8$^+$ T cells in the lymph node and the lung of irradiated recipient chimeric mice after reconstitution of bone marrow cells from the control (CD45.1$^+$) and the Cul4b^{cKO} (CD45.2$^+$) mice. Data from the three independent experiments is shown (n=18). Each dot represents a recipient mice and paired samples are connected with a dotted line. p-values were calculated using a paired two-tailed t-test **b, c)** The comparison of the naïve (CD62L^{hi}CD44^{lo}) and activated/effector memory like (CD62L^{lo}CD44^{hi}) CD8$^+$ T cells in the lymph nodes and lung of irradiated recipient chimeric mice after reconstitution. The line graphs show the relative ratios of naïve and activated CD8$^+$ T cells in the lymph nodes and lung of the recipient chimeric mice. Data from the three independent experiments is shown (n=18 recipient mice). Each paired sample is connected with a line. p-values were calculated using a paired two-tailed t-test. **d, e)** The line graph shows the relative frequencies of Ki-67$^+$CD8$^+$ T cells in the lung. Data is presented as line graph and each bar in the graph represents the mean value of two independent experiments with n=13 recipient per genotype. p-values were calculated using a paired two-tailed t-test. **f, g)** Each bar graphs show the percentages of naive (CD62L^{hi}CD44^{lo}), effector memory like (CD62L^{lo}CD44^{hi}) and central memory like (CD62L$^+$CD44^{hi}) CD8$^+$ T cells in the lymph nodes of recipient mice after reconstitution. Data is shown as the line graph from two independent experiments with (n=13 per genotype). Each paired sample is connected with a line and p-values were calculated using a paired two-tailed t-test. **h, i)** Representative plots of intracellular IFN-γ and TNF-α staining of control and Cul4b^{cKO} CD8$^+$ T cells within the same recipient mouse. **j, k)** Cytokine production in CD8$^+$ T cells stimulated with phorbol 12-myristate 13-acetate (PMA) and Ionomycin is shown. Data shown is from three independent experiments (n=18 recipient mice), bar represents the mean percentage of cytokine producing cells. Each paired sample is connected with a line and p-values were calculated using a paired two-tailed t-test. **l)** Bar graphs show the frequencies and numbers of DP (CD4$^+$CD8$^+$) cells in thymus. Data shown is from two independent experiments with n=4 for the control and n=5 for the Cul4b^{cKO} genotype and is represented as mean ± S.E.M. **m)** Flow cytometric analysis of thymocytes from control and Cul4b^{cKO} mice showing the TCR Vβ usage. Data is from 2 independent experiments with n=4 for the control and n=5 for the Cul4b^{cKO} groups and is shown as mean ± S.E.M. Source data are provided as a “Source Data” file

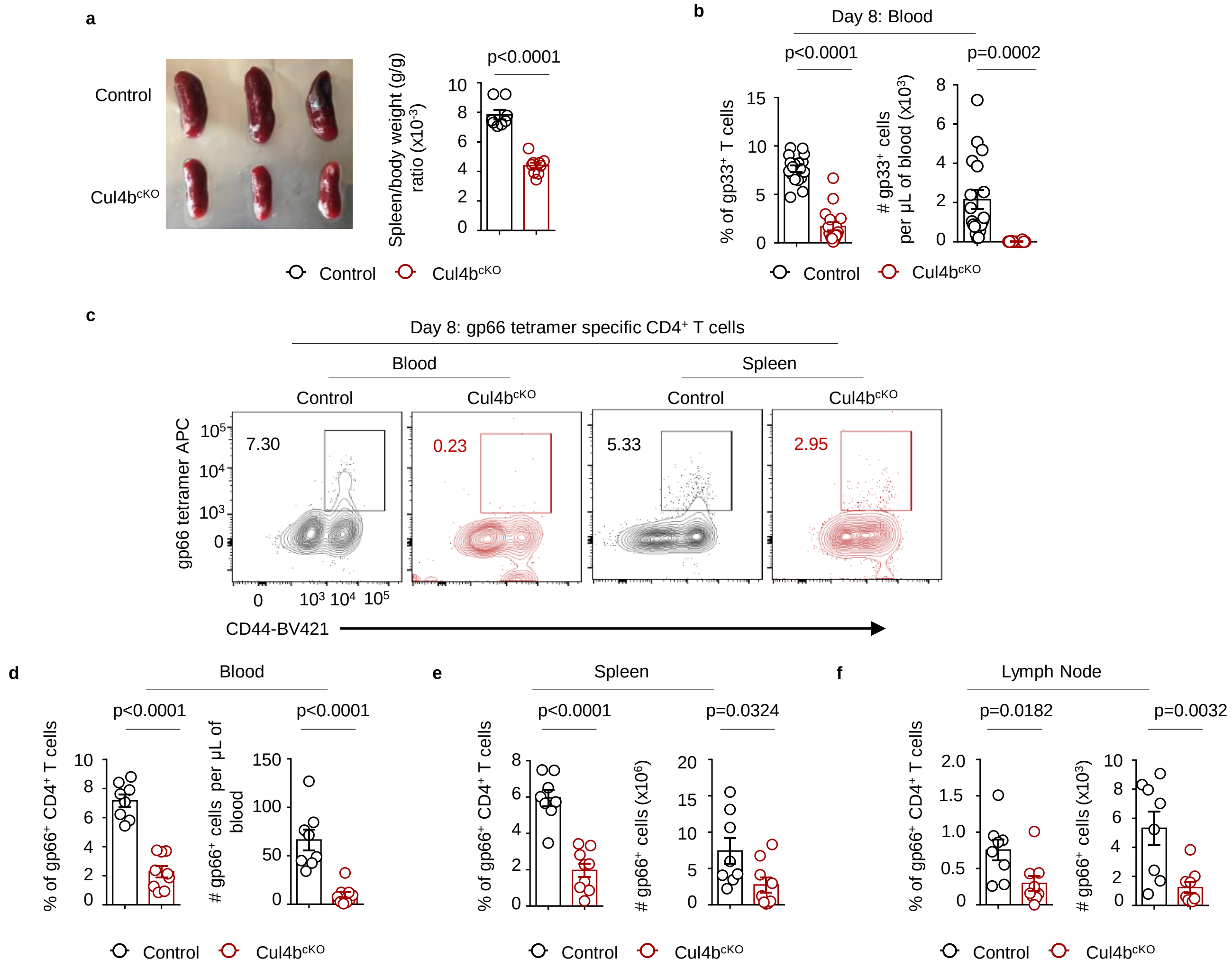

Supplementary Figure 3: Loss of Cul4b severely reduced the numbers of antigen specific CD8+ and CD4+ T cell responses following LCMV-arm infection: Cul4b^{cKO} and control mice (C57BL/6 background) were infected intraperitoneally with LCMV ($2{\times}10^5$ pfu). **a)** Spleen over body weight ratio of the control and Cul4b^{cKO} mice after LCMV infection is shown. Data is shown as mean ± S.E.M of n=8 control and n=9 Cul4b^{cKO} mice **b)** The percentages and numbers of gp33-specific CD8$^+$ T cells in the blood at d8 p.i. is shown as the mean ± S.E.M. Cumulative data of three independent experiments with n=18 for control and n=16 for Cul4b^{cKO} group is shown. p-values were calculated using an unpaired two-tailed t-test. **c)** Flow cytometry analysis of CD4$^+$ T cells showing percentages of gp66-specific cells at d8 p.i. in the blood and spleen. **d, e, f)** The percentages and numbers of gp66-specific CD4$^+$ T cells in the blood, spleen and lymph nodes is shown and is represented as mean ± S.E.M. Data is from two independent experiments (n=8 for the control and n=9 for the Cul4b^{cKO} group). p-values were calculated using an unpaired two-tailed t-test. Source data are provided as a “Source Data” file

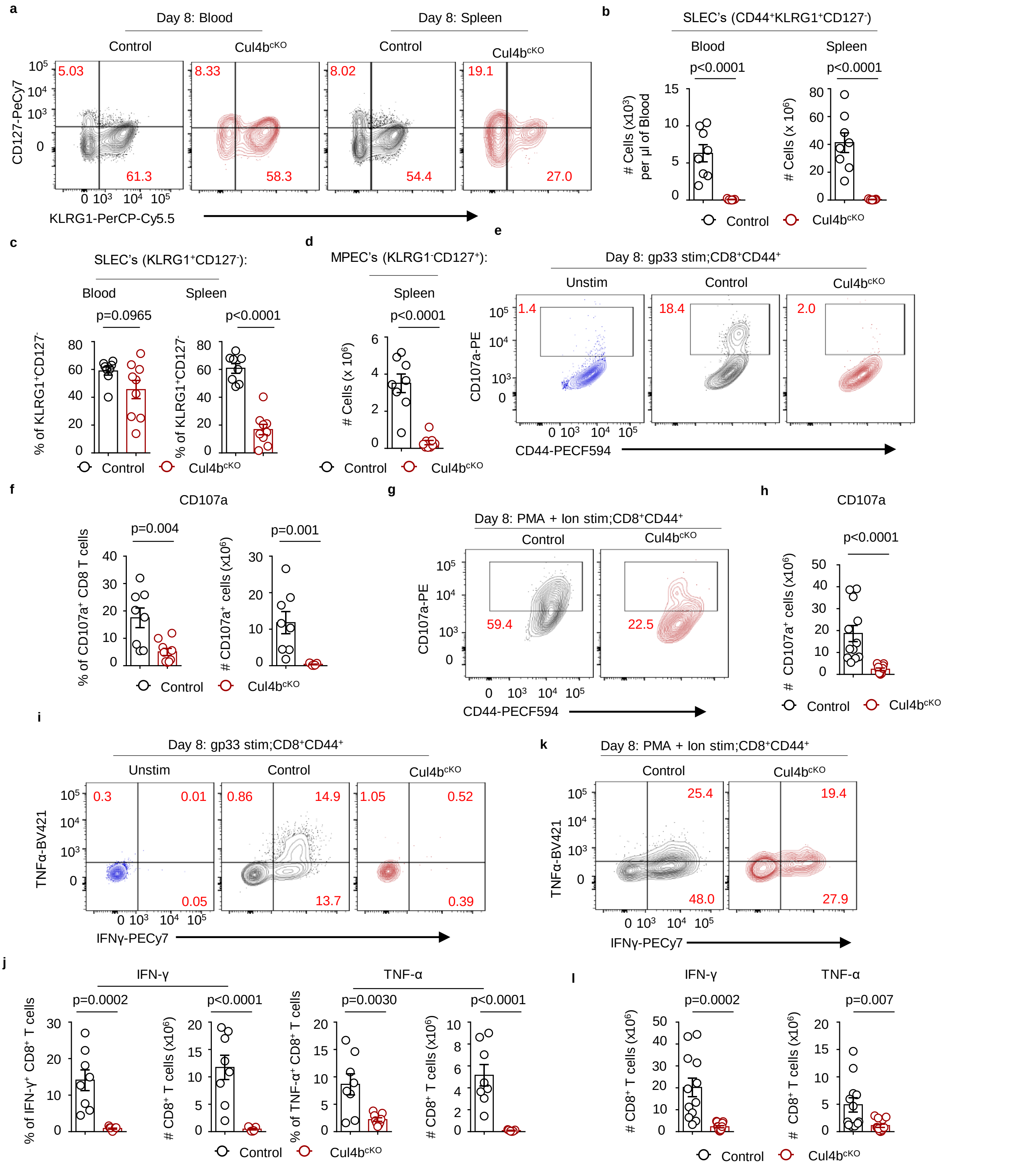

Supplementary Figure 4: Cul4b promotes $CD8^+$ T cell responses following LCMV infection a) Representative flow plots of the frequency of SLECs ($KLRG1^+CD127^-$) and MPECs ($KLRG1^-CD127^+$); gated on $CD8^+CD44^+$ T cells at day 8 post infection (p.i.) **b, c)**. Data shows the numbers and percentages of SLECs in the blood and spleen. Data from n=8 for the control and n=9 for the Cul4b^{cKO} groups is shown as mean ± S.E.M. p-values were calculated using an unpaired two-tailed t-test **d)** Data shows the numbers of MPECs in the spleen. Data from n=8 for the control and n=9 for the Cul4b^{cKO} genotypes is shown as mean ± S.E.M. p-values were calculated using an unpaired two-tailed t-test. **e, f)** Representative flow plots and summarized data showing CD107a expressing $CD8^+$ T cells in spleen cells harvested from mice at day 8 p.i. with LCMV and stimulated *ex vivo* with gp33-peptide for 4 h. Data from n=8 for control and n=9 for Cul4b^{cKO} genotypes is shown as mean ± S.E.M. p-values were calculated using an unpaired two-tailed t-test. **g, h)** Representative flow plots and summarized data showing CD107a expressing $CD8^+$ T cells in spleen cells harvested from mice at day 8 p.i. with LCMV and stimulated *ex vivo* with PMA and Ionomycin for 4 h. Data from n=12 control and n=13 Cul4b^{cKO} mice is shown as mean ± S.E.M. p-values were calculated using an unpaired two-tailed t-test **i, j)** Representative flow plots and summarized data shows the percentage and numbers of IFN-γ and TNF-α producing $CD8^+$ T cells from spleens harvested after day 8 p.i. with LCMV and stimulated *ex vivo* with gp33 peptide for 4 h. Data from n=8 control and n=9 Cul4b^{cKO} mice is shown as mean ± S.E.M. p-values were calculated using an unpaired two-tailed t-test **k, l)** Representative flow plots and summarized data showing percentages and numbers of IFN-γ and TNF-α producing $CD8^+$ T cells from spleens harvested after day 8 p.i. with LCMV and stimulated *ex vivo* with PMA and Ionomycin for 4 h. Data from n=12 control and n=13 Cul4b^{cKO} mice is shown as mean ± S.E.M. p-values were calculated using an unpaired two-tailed t-test. Source data are provided as a "Source Data" file

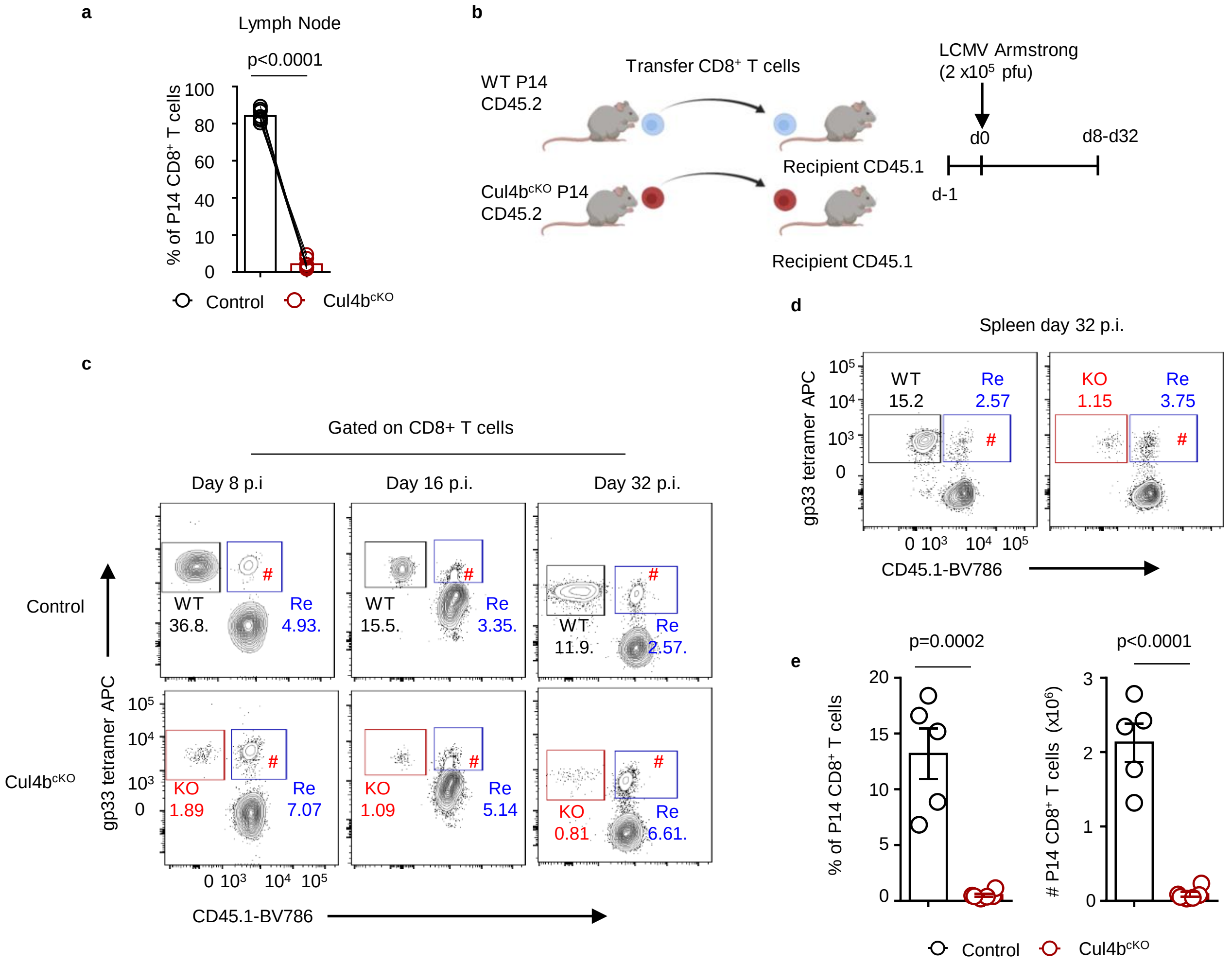

Supplementary Figure 5: Loss of Cul4b perturbs the earliest stages of effector differentiation a) Congenically distinct Cul4b^{cKO} and control P14 cells (total 5×10^4 ~ 1×10^5 cells) were mixed 1:1 and transferred into recipient mice. Recipient mice were intraperitoneally infected with LCMV-arm (2×10^5 pfu) and analyzed at the indicated time-points. Percentages of Cul4b^{cKO} and control P14 cells in the lymph nodes after day 8 post infection is shown. Data from n=8 recipient mice is shown and each paired sample is connected with a line. p-values were calculated using a paired two-tailed t-test **b)** Separate transfers of the Cul4b^{cKO} and control P14 cells (total 5×10^4 ~ 1×10^5 cells) into recipient mice. Recipient mice were infected intraperitoneally with LCMV (2×10^5 pfu) and analyzed at the indicated time-points. Schematic diagram was created with BioRender.com **c)** Representative flow plots show the show the percentages of donor P14 cells gated on CD8$^+$ T cells at d8, 16, 32 p.i. $^{\#}$Indicates gp33-specific CD8$^+$ T cells from the recipient mice. **d, e)** Representative flow plots and summarized data shows the percentages and numbers of P14 cells at d32 p.i. in spleen. Data are representative of two independent experiments (n=5 recipients were used for each genotype). Data is shown as mean ± S.E.M and p-values were calculated using an unpaired two-tailed t-test. $^{\#}$Indicates gp33-specific CD8$^+$ T cells from the recipient mice. Source data are provided as a "Source Data" file

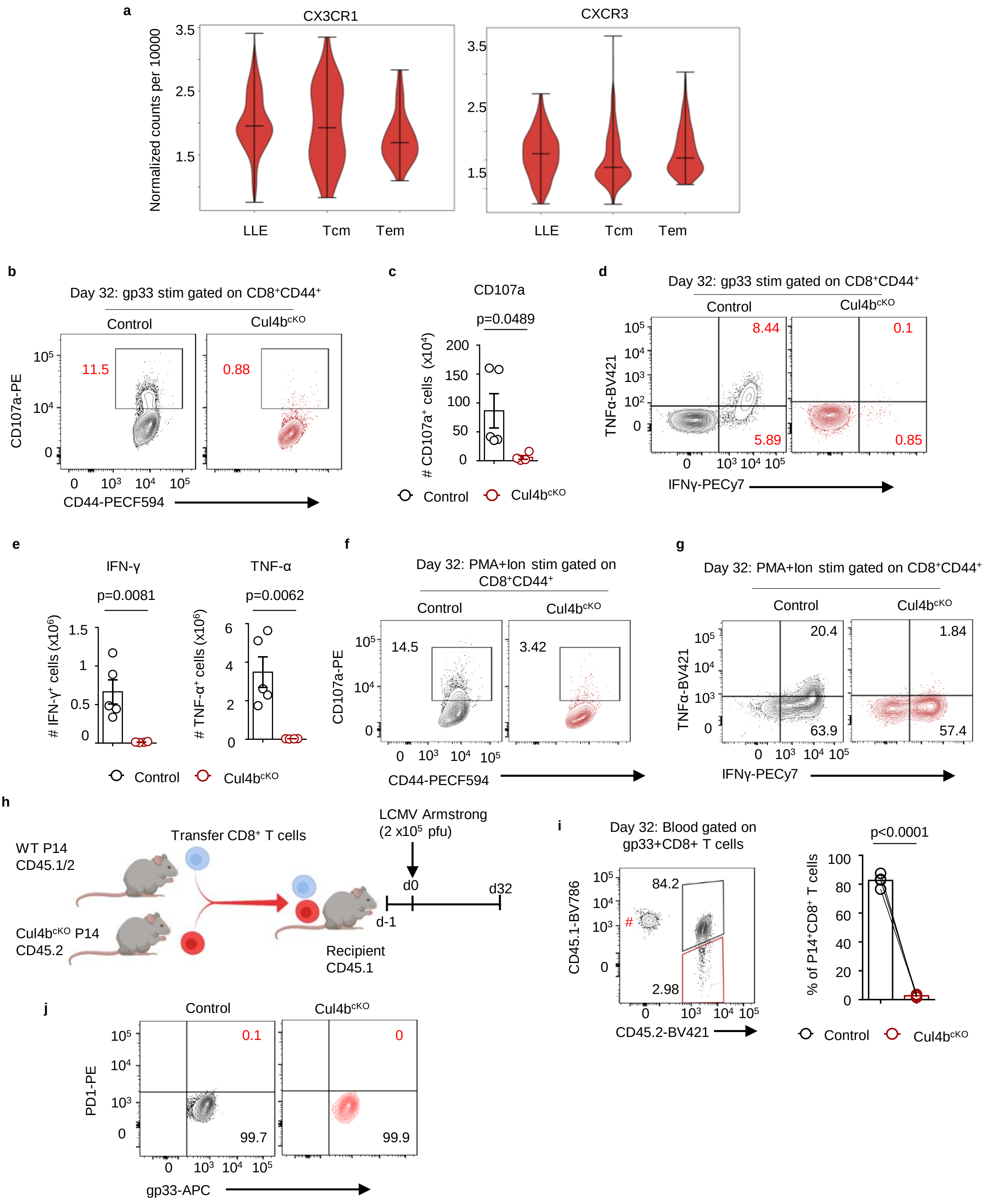

Supplementary Figure 6: Cul4b is essential for the generation of long-term immunity a) Bulk RNA-seq data from the subsets of long-lived effector cells (LLE;CD62L^{lo}IL-7Rα^{lo}), effector memory (T_{EM}; CD62L^{lo}IL-7Rα^{hi}), and central memory (T_{CM};CD62L^{hi}IL-7Rα^{hi}) cells. The subsets were sorted at D35 post infection. The gene-expression pattern of CX3CR1 and CXCR3 in LLE, TEM, and TCM is shown. The violin plot shows the density, range, median, and interquartile range (IQR) of the data **b, c, d, e)** Cul4b^{cKO} and control mice (C57BL/6 background) were infected intraperitoneally with LCMV (2×10^5 pfu) and analyzed at day 32 p.i. for the expression of CD107a, IFN-γ and TNF-α. Number of CD8$^+$ T cells expressing CD107a, IFN-γ and TNF-α isolated from LCMV infected the Cul4b^{cKO} and control mice is shown. CD107a, IFN-γ and TNF-α were analyzed after stimulation with gp33-peptide. Data from n=5 for the control and n=4 for the Cul4b^{cKO} group is shown as mean ± S.E.M. p-values were calculated using an unpaired two-tailed t-test. **f, g)** Representative flow plots showing CD8$^+$ T cells expressing CD107a, IFN-γ and TNF-α from LCMV infected Cul4b^{cKO} and control mice following stimulation with PMA and Ionomycin. **h)** Congenically distinct Cul4b^{cKO} and control P14 cells (total 5×10^4 ~ 1×10^5 cells) were mixed 1:1 and transferred to recipients. Recipient mice were infected intraperitoneally with LCMV (2×10^5 pfu) and analyzed at d32 to assess CD8+ memory T cells. Schematic diagram was created with BioRender.com. **i)** Flow cytometry plots of gated P14 cells showing percentages of wild-type (upper, CD45.1$^+$CD45.2$^+$) and Cul4b^{cKO} (lower, CD45.2$^+$) at adoptive transfer and at d32 p.i. in the spleen. #Indicates P14 cells from the recipient mice. Percentages of Cul4b^{cKO} and control P14 cells in the blood at day 32 p.i is shown and each paired sample (n=4) is connected with a line. p-values were calculated using a paired two-tailed t-test. **J)** Expression of PD-1 on congenically distinct Cul4b^{cKO} and control P14 cells at day 32 post infection. Flow plot shows the representative data. Source data are provided as a "Source Data" file

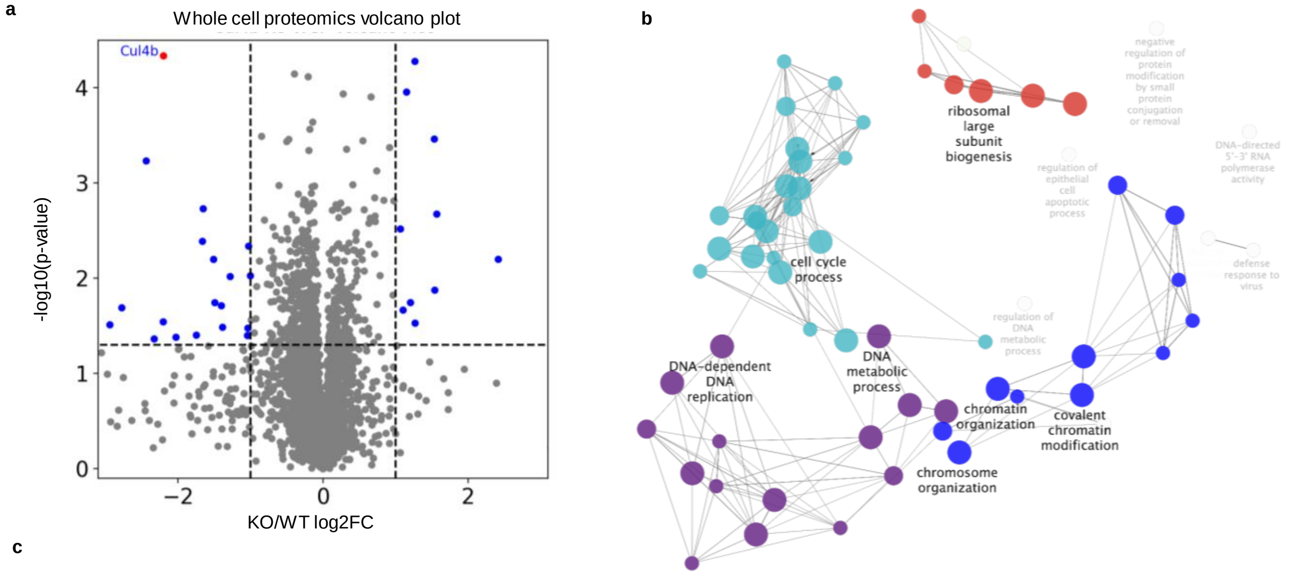

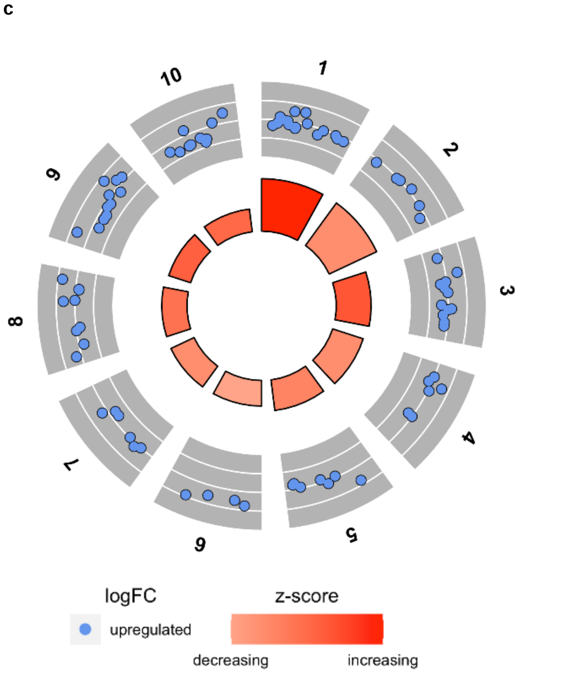

No.	ID	Description
1	GO:0007049	Cell Cycle
2	GO:0006270	DNA Replication Initiation
3	GO:0051301	Cell Division
4	GO:0000082	G1/S Transition of Mitotic cell Cycle
5	GO:0006260	DNA Replication
6	GO:0006268	DNA unwinding involved in DNA Replication
7	GO:0042254	Ribosome Biogenesis
8	GO:0051726	Regulation of Cell Cycle
9	GO:0006974	Cellular response to DNA Damage Stimulus
10	GO:0016567	Protein Ubiquitination

Supplementary Figure 7: Cul4b controls cell cycle and DNA damage response pathways: a) Cul4b^{cKO} and control CD8$^+$ T cells were stimulated for 40 h with anti-CD3/CD28 mAbs (5 µg/mL). Proteins were quantified by iBAQ intensities and compared between Cul4b^{cKO} and control CD8$^+$ T cells to generate fold changes. Volcano plot shows the differentially regulated proteins, blue dots indicate top most differentially regulated proteins ($p < 0.05$, $n = 3$). Proteins with higher abundance in Cul4b^{cKO} CD8$^+$ T cells are on the right side of the plot and those in control CD8$^+$ T cells are on the left side. Notably, Cul4b was one the most differentially abundant proteins and is represented as red dot for emphasis. **b)** ClueGO was used to integrate GO terms with KEGG pathways to create a functionally organized GO/pathway term network. Functionally grouped networks of enriched categories were generated for the differentially abundant proteins. GO terms are represented as nodes, and the node size represents the enrichment significance. Functionally related groups partially overlap and terms that were not grouped are shown in white. **c)** Ontological analysis of the upregulated proteins in Cul4b^{cKO} CD8$^+$ T cells was done by GOPlot. The circular plot depicts the enriched functional networks of proteins. The outer circle is a scatter plot for each biological term of the logFC of the enriched proteins. Within each network, a protein is depicted as a blue dot. The size of the inner trapezoids corresponds to the adjusted p-value and the color indicates the z-score as calculated by GOplot algorithm. p-values were calculated using two-tailed Fisher's exact tests, corrected for multiple comparisons via Bonferroni correction. Source data are provided as a “Source Data” file

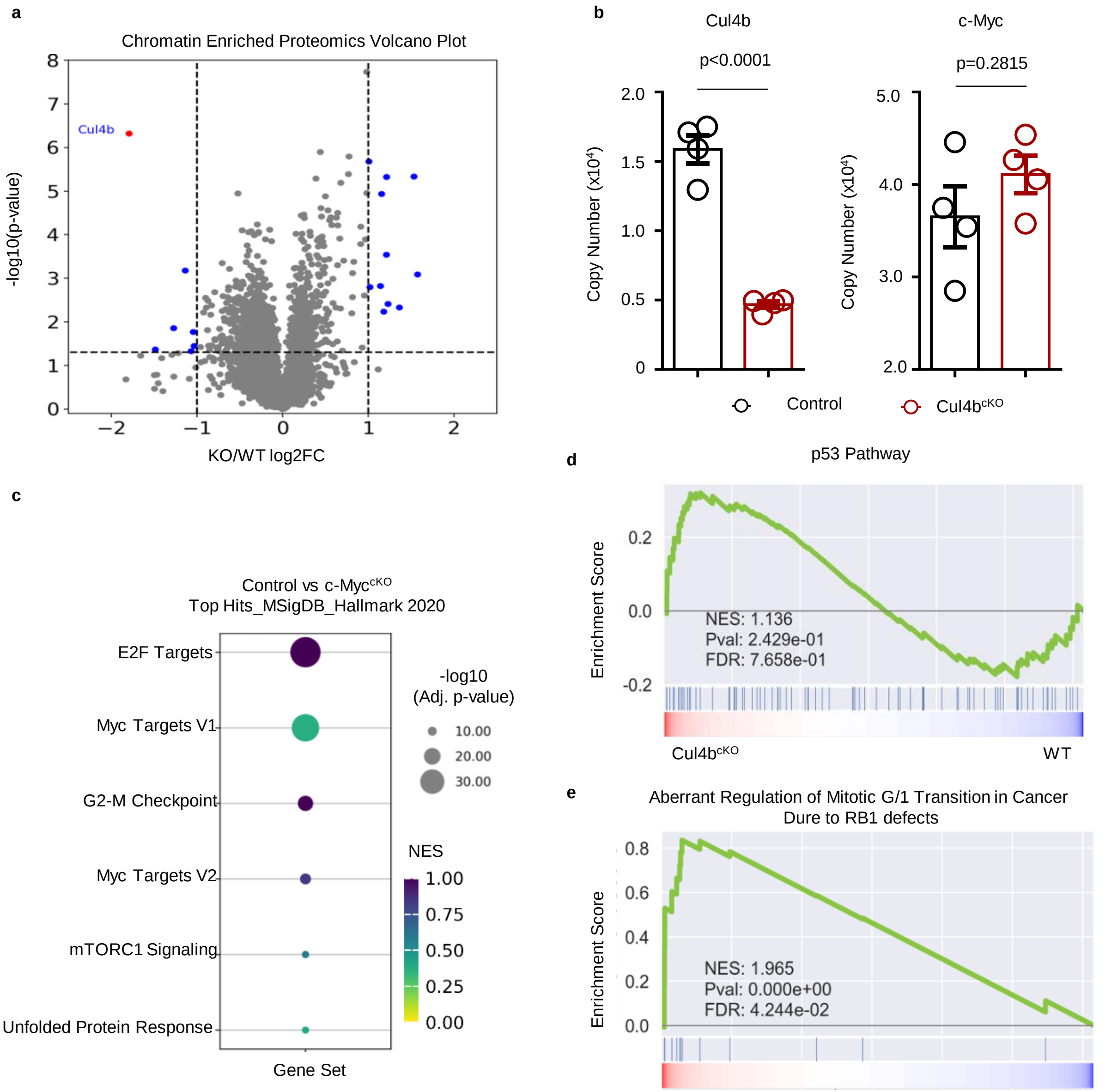

Supplementary Figure 8: c-Myc and Cul4b deleted CD8⁺ T cells share hallmark gene sets: a) Cul4b^{cKO} and control CD8$^+$ T cells were stimulated for 40 h with anti-CD3/CD28 mAbs (5µg/mL), and for the final 2 h in the culture Camptothecin (2 µM) was added. Chromatin enriched proteins were quantified by iBAQ intensities and these were compared between Cul4b^{cKO} and control CD8$^+$ T cells to generate fold changes. Volcano plot show the differentially abundant proteins, blue dots indicate top most differentially regulated proteins (p <0.05, *n* = 4). p-values were calculated using two-tailed Fisher's exact tests, corrected for multiple comparisons via Bonferroni correction. **b)** Quantification of protein abundances of Cul4b and c-Myc in CD8$^+$ T cells using mass spectrometry. The copy numbers of Cul4b and c-Myc in TCR activated (40 h) CD8$^+$ T cells is shown and was calculated using the proteomic ruler method. Data is shown as mean ± S.E.M and p-values were calculated using an unpaired two-tailed t-test **c)** Hallmark gene sets were assessed among the differentially abundant proteins between the control and c-Myc^{cKO} CD8$^+$ T cells. E2F targets, G2-M checkpoint, c-Myc-targets V1/V2 were the most significantly enriched in c-Myc^{cKO} CD8$^+$ T cells. The G2-M checkpoint, c-Myc-targets V1/V2 gene sets were also enriched in Cul4b^{cKO} CD8$^+$ T cells. The size of bubble represents an adjusted p-value while the green-blue color scale represents the normalized gene enrichment score. **d)** GSEA performed on hallmark gene sets of the p53 pathway showed its enrichment in Cul4b^{cKO} CD8$^+$ T cells over control CD8$^+$ T cells. **e)** GSEA performed on the Reactome term “Aberrant Regulation of Mitotic G/1 Transition in Cancer due to RB1 defects” shows an enrichment in Cul4b^{cKO} CD8$^+$ T cells over control CD8$^+$ T cells. p-values were calculated by two-tailed Fisher's exact tests and were corrected for multiple comparisons via the Benjamini-Hochberg procedure. Normalized enrichment score (NES) reflects the degree to which the gene set is overrepresented at the top or bottom of a ranked list of genes, normalized for gene set size and correlations between the gene set and expression dataset. NES was calculated via GSEA. Source data are provided as a “Source Data” file

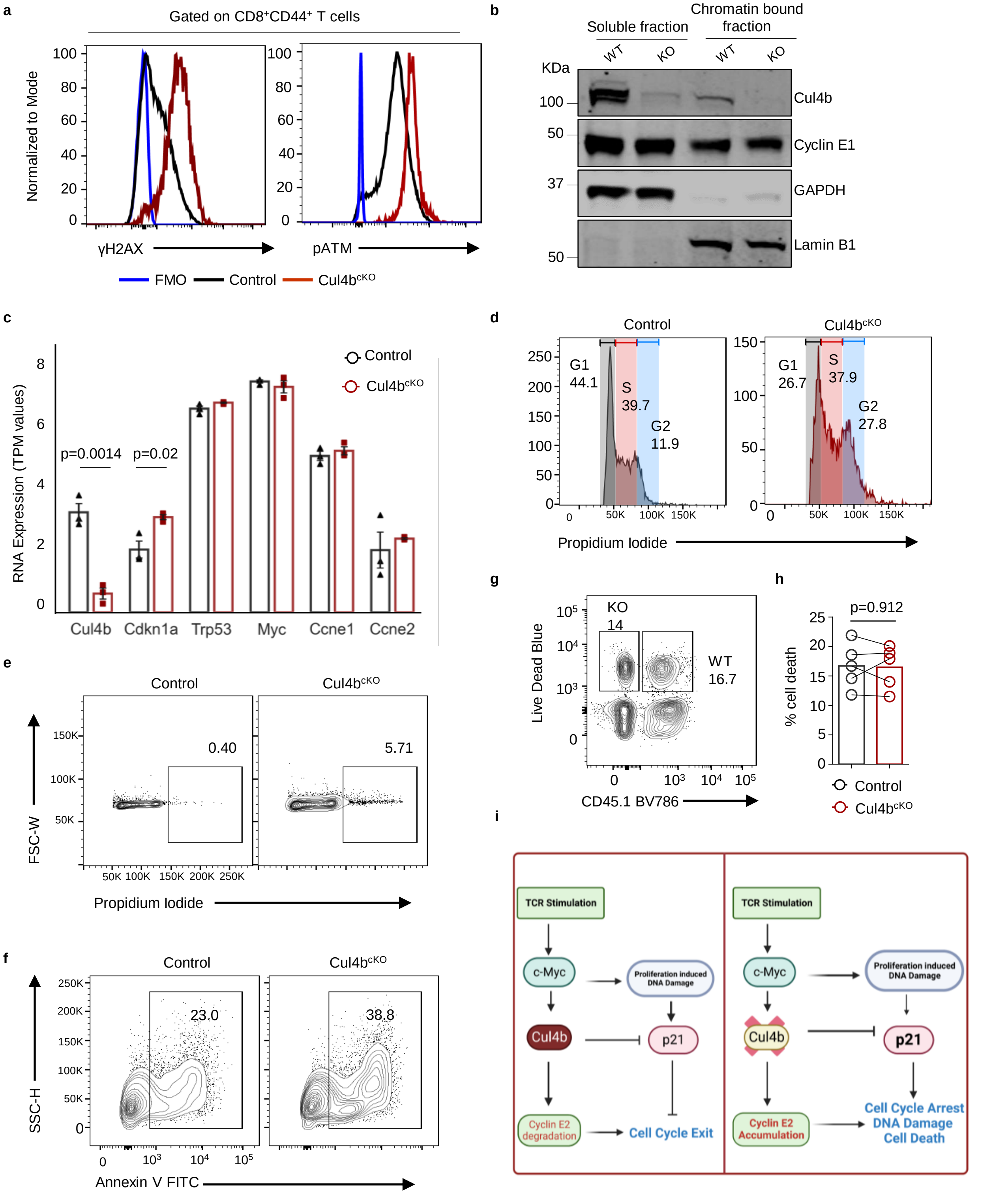

Supplementary Figure 9: Cul4b maintains genome integrity of proliferating CD8$^+$ T cells. a) Activated CD8$^+$ T cells from control and Cul4b^{cKO} mice infected with LCMV were analyzed for the expression of γ-H2AX and pATM. Fluorescence minus one (FMO) controls were used to interpret the data. **b)** Cyclin E1 in Cul4b^{cKO} and control CD8$^+$ T cells stimulated for 40 h with anti-CD3/CD28 mAbs (5 μg/mL). Soluble and chromatin-bound proteins were fractionated and analyzed by immunoblot. Lamin B1 and GAPDH were used to assess enrichment of chromatin bound and soluble fractions, respectively. Data is representative of three independent experiments. **c)** Bar graph showing the transcript levels of *Cul4b, p53, p21, c-Myc, Ccne1* and *Ccne2* in the control and Cul4b^{cKO}CD8$^+$ T cells that were stimulated with anti-CD3/CD28 mAbs (5 μg/mL) for 24 h. Data is shown as mean ± S.E.M and p-values were calculated using an unpaired two-tailed t-test **d)** Cell cycle analysis was assessed using PI staining and flow cytometry, flow plots show the percentages of cells in G1, S, and G2/M phases from the control and Cul4b^{cKO} CD8$^+$ T cells. **e)** Re-replication was assessed using PI staining and flow cytometry, flow plots shows the percentages of cells with DNA greater than 4n in the control and Cul4b^{cKO} CD8$^+$ T cells. **f)** Naive CD8$^+$ T cells were stimulated with anti-CD3 and CD28 mAbs (5μg/ml) for 3 days to allow multiple rounds of cell division. After day 3 cells were stained with Annexin V-FITC antibody. The percentage of apoptotic cells (Annexin-V$^+$) were analyzed by flow cytometry. **g, h)** Naïve CD8$^+$ T cells isolated from control (CD45.1) and Cul4b^{cKO} (CD45.2) mice were co-cultured and stimulated in vitro with anti-CD3/CD28 mAbs (5μg/mL) for 3 days in the presence of Rapamycin. The relative proportions of the dead cells were assessed by flow cytometry. Data is representative of two independent experiments (n=5) and is shown as mean ± S.E.M. **i)** Upon TCR ligation, c-Myc increases the levels of active Cul4b. Cul4b enters the nucleus and regulates levels of Cyclin E2 and p21 to aid cell cycle progression and avoid replication stress. In absence of Cul4b, CD8$^+$ T cells accumulated high levels of p21 and Cyclin E2 fueling replication stress, and triggering genomic instability and apoptosis. Diagram was created with BioRender.com. Source data are provided as a "Source Data" file

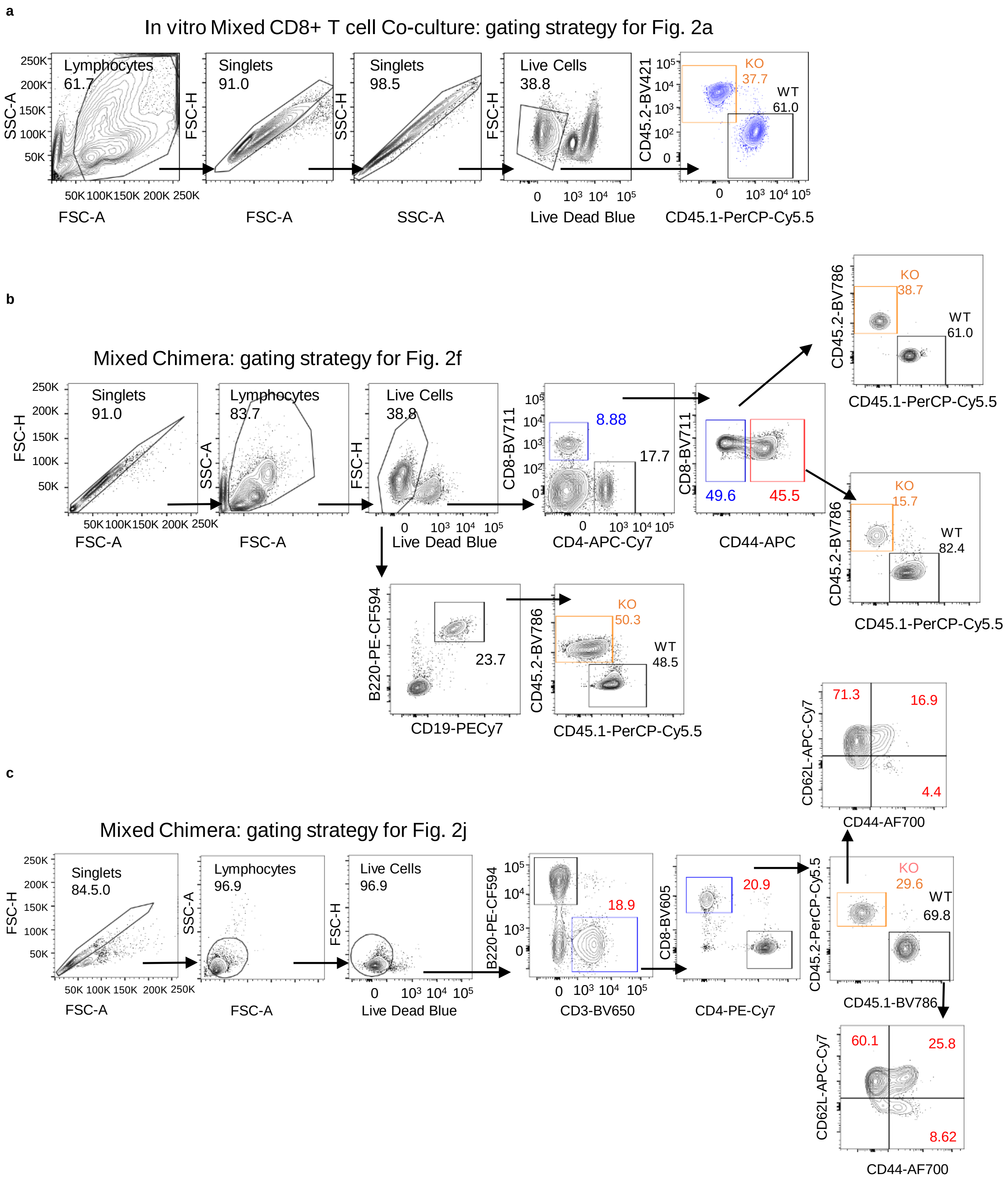

Supplementary Figure 10: Gating Strategy used to analyze the data

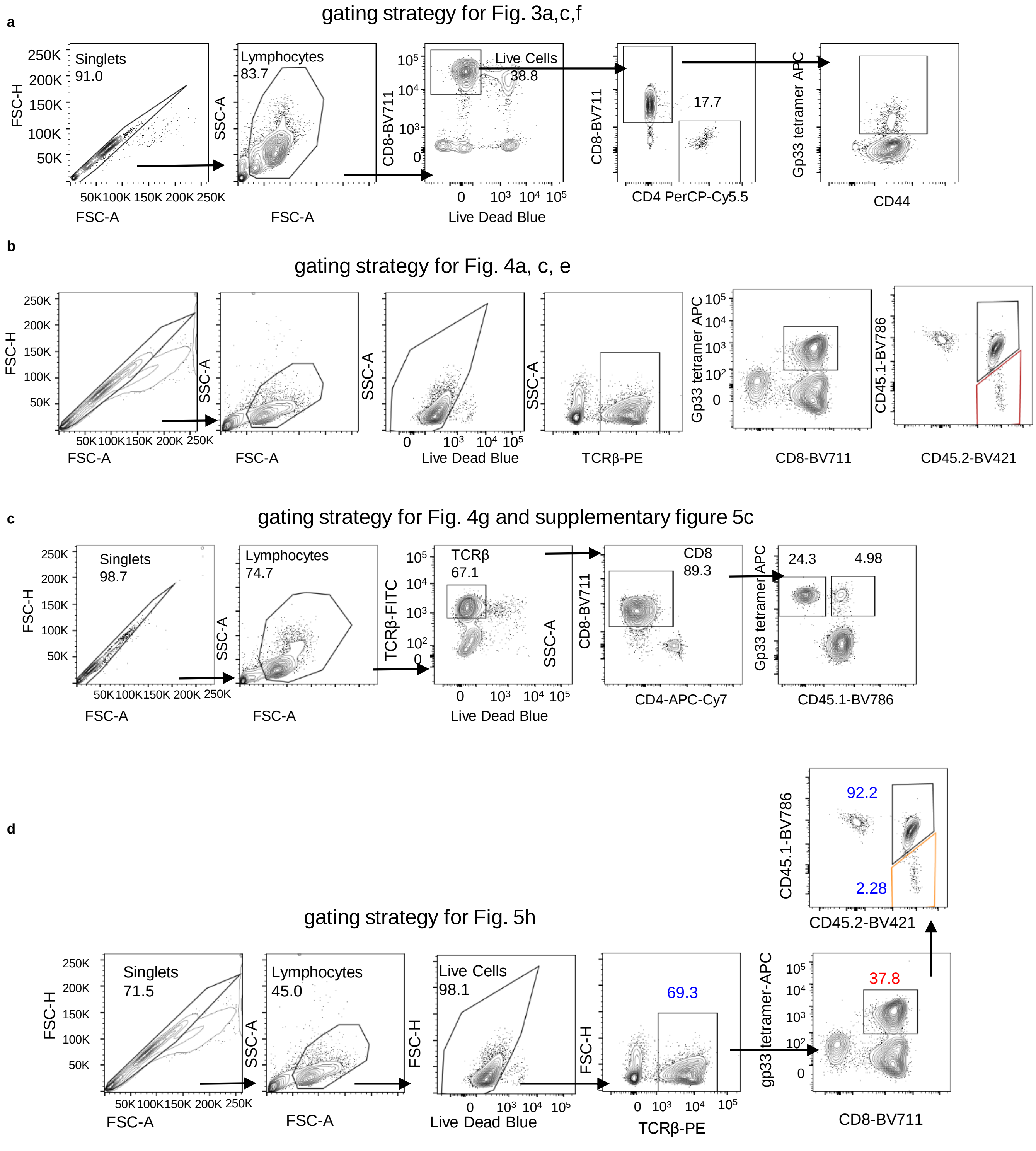

Supplementary Figure 11: Gating Strategy used to analyze the data
