# Supplementary material for: c-Myc uses Cul4b to preserve genome integrity and promote antiviral CD8+ T cell immunity
Source: Nat Commun. 2023 Nov 4;14:7098. doi: 10.1038/s41467-023-42765-7 (PMC10625626; doi:10.1038/s41467-023-42765-7)
Supplement: Supplementary file 3 — Reporting Summary [file 41467_2023_42765_MOESM3_ESM.pdf]

Corresponding author(s): Dr. Asif Dar
Dr. Paula Oliver

Last updated by author(s): Sep 21, 2023

Reporting Summary

Nature Portfolio wishes to improve the reproducibility of the work that we publish. This form provides structure for consistency and transparency in reporting. For further information on Nature Portfolio policies, see our Editorial Policies and the Editorial Policy Checklist.

Statistics

For all statistical analyses, confirm that the following items are present in the figure legend, table legend, main text, or Methods section.

n/a	Confirmed	
☐	☒	The exact sample size (n) for each experimental group/condition, given as a discrete number and unit of measurement
☒	☐	A statement on whether measurements were taken from distinct samples or whether the same sample was measured repeatedly
☐	☒	The statistical test(s) used AND whether they are one- or two-sided<br>*Only common tests should be described solely by name; describe more complex techniques in the Methods section.*
☒	☐	A description of all covariates tested
☒	☐	A description of any assumptions or corrections, such as tests of normality and adjustment for multiple comparisons
☐	☒	A full description of the statistical parameters including central tendency (e.g. means) or other basic estimates (e.g. regression coefficient) AND variation (e.g. standard deviation) or associated estimates of uncertainty (e.g. confidence intervals)
☐	☒	For null hypothesis testing, the test statistic (e.g. F, t, r) with confidence intervals, effect sizes, degrees of freedom and P value noted<br>*Give P values as exact values whenever suitable.*
☒	☐	For Bayesian analysis, information on the choice of priors and Markov chain Monte Carlo settings
☒	☐	For hierarchical and complex designs, identification of the appropriate level for tests and full reporting of outcomes
☐	☒	Estimates of effect sizes (e.g. Cohen's d, Pearson's r), indicating how they were calculated

Our web collection on statistics for biologists contains articles on many of the points above.

Software and code

Policy information about availability of computer code

Data collection	BD LSR FORTESSA WITH FACSDiva Software, ImageStudio Ver 5.2, Evos FL Auto epifluorescence microscopy
Data analysis	All flow cytometry data was analyzed using FLowJo V10 10 (Tree Star)<br>All stastical tests were run using Graphpad Prsim 7 and 9,<br>ImageStudio Lite ver 5.2, Microsoft Excel, R studio, Image J (1.53k), Cytoscape (v. 3.9.1), DAVID, gseapy package (v.1.0.4) in Python, Integrated Genome Browser (IGB; v.9.1.6), Python (v. 3.9.13) , ScanPy package (v. 1.9.1) in Python., AnnData package (v. 0.8.0) in Python

For manuscripts utilizing custom algorithms or software that are central to the research but not yet described in published literature, software must be made available to editors and reviewers. We strongly encourage code deposition in a community repository (e.g. GitHub). See the Nature Portfolio guidelines for submitting code & software for further information.

Data

Policy information about availability of data

All manuscripts must include a data availability statement. This statement should provide the following information, where applicable:

- Accession codes, unique identifiers, or web links for publicly available datasets
- A description of any restrictions on data availability
- For clinical datasets or third party data, please ensure that the statement adheres to our policy

The mass spectrometry proteomics data has been deposited to the ProteomeXchange Consortium via the PRIDE 69 partner repository with accession number

PXD041220 [https://proteomecentral.proteomexchange.org/cgi/GetDataset?ID=PXD041220]. The bulk-RNA-seq data have been deposited in the GEO repository under accession codes GSE228695 [https://www.ncbi.nlm.nih.gov/geo/query/acc.cgi?acc=GSE228695]. The publicly available datasets with accession numbers GSE131847 [https://www.ncbi.nlm.nih.gov/geo/query/acc.cgi?acc=GSE131847], GSE58081 [https://www.ncbi.nlm.nih.gov/geo/query/acc.cgi?acc=GSE58081], GSE183619 [https://www.ncbi.nlm.nih.gov/geo/query/acc.cgi?acc=GSE183619], PXD004140 [https://proteomecentral.proteomexchange.org/cgi/GetDataset?ID=PXD004140], PXD016105 [https://proteomecentral.proteomexchange.org/cgi/GetDataset?ID=PXD016105], PXD012058 [https://proteomecentral.proteomexchange.org/cgi/GetDataset?ID=PXD012058] were used in this study.
All other data supporting the findings are available within this paper and supplementary information. Source data are provided with this paper

Research involving human participants, their data, or biological material

Policy information about studies with human participants or human data. See also policy information about sex, gender (identity/presentation), and sexual orientation and race, ethnicity and racism.

Reporting on sex and gender	NA
Reporting on race, ethnicity, or other socially relevant groupings	NA
Population characteristics	NA
Recruitment	NA
Ethics oversight	NA

Note that full information on the approval of the study protocol must also be provided in the manuscript.

Field-specific reporting

Please select the one below that is the best fit for your research. If you are not sure, read the appropriate sections before making your selection.

☒ Life sciences ☐ Behavioural & social sciences ☐ Ecological, evolutionary & environmental sciences

For a reference copy of the document with all sections, see nature.com/documents/nr-reporting-summary-flat.pdf

Life sciences study design

All studies must disclose on these points even when the disclosure is negative.

Sample size	No pre experiemntal statistical methods, randomization or blinding were used in animal experiments. The size of the group and number of replicates was determined by the magnitude of the effect being observed and sample to sample variation in the parameters being measured. Sample size were deemed sufficient based on the reproducibility between independent experiments. Mice of both sexes were used for experiments.<br>For adoptive T cell transfer experiments and mix bone marrow chimera experiments sample size was determine based on prior experience and published literature (PMID: 24584090; PMID: 33524014; PMID: 35589717). For time point experiments more than 4 mice per group were used.<br>For RNASeq at least three biological replicates were used for each group<br>For whole cell proteomics three biological replicates while for chromatin enriched mass spectrometry four biological replicated were used for each group PMID: 33524014; PMID: 31061531
Data exclusions	No data were excluded. Occasionally flow data that showed staining issue or immunoblotting data that showed prominent non specific bands near the protein of interest were were excluded. For example in case of p21 in chromatin bound fraction, non specific bands hindered analysis
Replication	Results were confirmed in at least two independent experiments, unless otherwise indicated in figure legends.
Randomization	Litter mate controls were used in most experiments and randomly assigned to groups. In certain experiments, CD45.1 or CD45.1/CD45.2 wild type mice were used. In all experiment animals were gender matched and age matched.
Blinding	Blinding was not performed due to requirements for cage labeling and mouse labelling, and data analysis was strictly quantitative and not objective

Reporting for specific materials, systems and methods

We require information from authors about some types of materials, experimental systems and methods used in many studies. Here, indicate whether each material, system or method listed is relevant to your study. If you are not sure if a list item applies to your research, read the appropriate section before selecting a response.

Materials & experimental systems

n/a	Involved in the study
☐	☒ Antibodies
☒	☐ Eukaryotic cell lines
☒	☐ Palaeontology and archaeology
☐	☒ Animals and other organisms
☒	☐ Clinical data
☒	☐ Dual use research of concern
☒	☐ Plants

Methods

n/a	Involved in the study
☐	☒ ChIP-seq
☐	☒ Flow cytometry
☒	☐ MRI-based neuroimaging

Antibodies

Antibodies used

Following antibodies TCRβ [Clone H57-597,catalog numbers 109208 and109205;1:300], CD3 [Clone 17A2, 145-2C11, catalog numbers 100237and 100328;1:300], CD4 [Clone GK1.5 and RM4-5, catalog numbers 100545, 100406, 100510, 100434,100422;1:300], CD8a [Clone 53-6.7, catalog numbers 100743, 100738, 100751, 100706, 100714, 100722, 100712, 100730;1:300], CD19 [Clone 6D5 catalog numbers 115543, 115520;1;300], CD44 [Clone IM7, catalog numbers 103020, 103006, 103056, 103026, 103027, 103012;1:300], CD45.1 [Clone A20, catalog numbers 110713, 110716, 110728, 110737, 110731; 1:300], CD45.2 [Clone 104, catalog numbers 109824, 109830, 109820, 109828;1:300], B220 [Clone RA3-6B2, catalog number 103206;1:300], CD62L [Clone MEL-14 catalog number 104448, 104428; 1:300], CD90.2 [Clone 30-H12 catalog numbers 105306, 105319, 105327; 1:300], CD107a [Clone 1D4B, catalog number 121611; 1:100], CD127 [Clone A7R34, catalog number 135039; 1:200], KLRG1 [Clone 2F1, catalog number 138429; 1:300], TNF-α [clone MP6-XT22, catalog numbers 506308 and 506318; 1:200], IFN-γ [Clone XMG1.2, catalog number 505826; 1:200], ATM phospho Ser1981 [Clone 10H11.E12, catalog number 651203; 1:100], H2A.X phospho Ser139 [Clone 2F3, catalog number 613407; 1:100], CD62L (Clone MEL-14, catalog number 104448, 104428; 1:200], I, Ki-67 [1Clone 6A8 catalog number 652411;1:200] from BioLegend.

TCR Vβ Screening Panel catalog number 557004 (Vβ2 [B20.6;1:100], Vβ4 [KT4:1:100], Vβ5 [MR9-4;1:100], Vβ6 [RR4-7;1:100], Vβ7 [TR310;1:100], Vβ8 [MR5-2;1:100] and Vβ17 [KJ23;1:100]) from BD Bioscience .
PD-1 [Clone J43 catalog number 11-9985-81; 1:300], KLRG1 [Clone 2F1, catalog numbers 46-5893-80, 25-5893-80, 17-5893-81;1:300] CD127 [A7R34, catalog numbers 17-1271-82 and 47-1271-80; 1:200] from eBiosciences.
Annexin V [Lot number 2354351, catalog number 640906] from invitrogen
MHC class I H-2Db gp33 tetramers (1:200) and MHC class II I-Ab gp66 tetramers (1:200) were obtained from the National Institute of Health Tetramer Core.

For western blotting following primary antibodies Cul4b (Sigma Aldrich; lot number B10150; catalog number HPA011880 1:500) and Cul4b (ProteinTech, catalog number 12916-1-AP; 1:1000), p53 [Abcam, clone pab122, catalog number ab90363; 1:1000), Lamin B1 (Cell Signaling Technology, Clone D4Q4Z, catalog number 12586S; 1:1000), c-Myc (Cell Signaling Technology, Clone D84C12 , catalog number 5605S ; 1:1000), Cyclin E1 (Santa Cruz; Clone M-20, catalog number sc-4811:500; 1:500), Cyclin E2 (Cell Signaling Technology, Lot number 3, catalog number 4132S; 1:1000), p21 (Santa Cruz; clone F-5, catalog number sc-6246; 1:200), GAPDH (Millipore, Lot number 2910381, catalog number MAB374; 1:5000) and β-actin (Santa Cruz, Clone C4, catalog number, sc-47778; 1:5000) were used. Immunostaining was performed using appropriate goat anti-mouse (Invitrogen, lot number XA336885, catalog number A32730; 1:5000) and goat anti-rabbit (Invitrogen, lot number2260898 catalog number A21109) secondary antibodies.

Validation

Antibodies used in this study are commercially available. Validation of individual antibodies has been performed by respective manufactures. Antibodies were validated on C57BL/6 splenocytes and were quality tested for flow cytometry assay. In case of CD107a, IFN-γ and TNF-α C57BL/6 splenocytes were activated in presence of brefeldin A and quality tested for intracellular flow cytometry assay. Further all conjugated antibodies used for flow cytometry were validated before use and representative flow plots panels are shown in Fig 2, 3, 4, 5, 7 and supplementary data fig, 2, 3, 4, 5, 6. For γ-H2AX and pATM antibodies fluorescence minus one (FMO) controls were used (supplementary fig. 9a). For CD107a, IFN-γ and TNF-α antibodies unstimulated controls were used to validate the positive staining as shown in supplementary figure 4e, 4i.
For western blot experiments we have detected proteins at appropriate molecular weight which was consistent with the molecular weight shown in vendor(s) website and its predicted molecular weight based on the amino acid sequence. The Cul4b antibody was validated using the protein samples derived from control and Cul4b- deficient CD8+ T cells as shown in Supplementary figure 1h and Fig. 7a. Cul4b antibody was also validated by Immunoprecipitation followed by mass-spectrometry in our PLOS Biology Manuscript (PMID 33524014). p53 has been validated for western blotting on HCT-116 cell lysate, A431 cells, rat brain tissue extract, mouse brain tissue extract by the vendor and we used camptothecin treated T cells as a positive control for its induction Fig. 7a. Western blot analysis of p21 Waf1/Cip1 expression was shown in Cyclopamine treated SW480 whole cell lysates and after treatement p21 levels increased. We used camptothecin treated T cells as a positive control for its upregulation Fig. 7a and source fig 7a data. c-Myc has been validated on the for western blot analysis on the extracts from control HEK293 cells or c-Myc knockout HEK293 cells by vendor. Cyclin E1 has been validated for western blotting analysis on A549 cells [PMID 23543735].
For Cyclin E2 Western blot analysis, extracts from MCF-7, SK-N-MC and HeLa cells, untreated or treated with the proteasome inhibitor MG-132 were used by the vendor.

Animals and other research organisms

Policy information about studies involving animals; ARRIVE guidelines recommended for reporting animal research, and Sex and Gender in Research

Laboratory animals	C57BL/6J (B6), B6.CD45.1, Rag1−/−, and CD4-Cre mice were from the Jackson Laboratory. The mice used were kept in a C57BL/6J (B6) background. Cul4bfl/fl mice was generated using CRISPR/Cas9 as described previously (Dar, Sawada et al. 2021). For some of the experiments, Cul4bfl/fl mice were crossed to P14 TCR transgenic mice. All mice were bred in house under specific pathogen-free conditions in the animal facility at the Children's Hospital of Philadelphia (CHOP). The mice were housed at 18-23°C with 40-60% humidity, with 12-hr light and 12-hr dark cycles. All mice, if not specifically mentioned in this manuscript, were 6-12 weeks of age, and both sexes were used without randomization or blinding.
Wild animals	No wild animals were used in the study
Reporting on sex	Both sexes were used in this study without randomization or blinding.
Field-collected samples	No field collected samples were used in the study
Ethics oversight	Animal housing, care, and experimental procedures were performed in compliance with the CHOP Institutional Animal Care and Use Committee

Note that full information on the approval of the study protocol must also be provided in the manuscript.

Plants

Seed stocks	NA
Novel plant genotypes	NA
Authentication	NA

ChIP-seq

Data deposition

☒ Confirm that both raw and final processed data have been deposited in a public database such as GEO.

☐ Confirm that you have deposited or provided access to graph files (e.g. BED files) for the called peaks.

Data access links<br>*May remain private before publication.*	CD8+ T cell anti-c-Myc and input ChIP-seq BedGraph data mapped to the mm9 genome were obtained from GSE58081(Chou, Pinto et al. 2014)
Files in database submission	N/A; ChIP-seq from public repository (see GSE58081 for further details)
Genome browser session<br>(e.g. UCSC)	N/A; ChIP-seq from public repository (see GSE58081 for further details)

Methodology

Replicates	N/A; ChIP-seq from public repository
Sequencing depth	N/A; ChIP-seq from public repository
Antibodies	N/A; ChIP-seq from public repository
Peak calling parameters	N/A; ChIP-seq from public repository
Data quality	N/A; ChIP-seq from public repository
Software	Integrated Genome Browser (IGB; v.9.1.6) was used to analyze peaks from the bedGraph provided by the original authors.

Flow Cytometry

Plots

Confirm that:

☒ The axis labels state the marker and fluorochrome used (e.g. CD4-FITC).

☒ The axis scales are clearly visible. Include numbers along axes only for bottom left plot of group (a 'group' is an analysis of identical markers).

☒ All plots are contour plots with outliers or pseudocolor plots.

☒ A numerical value for number of cells or percentage (with statistics) is provided.

Methodology

Sample preparation	Lymph nodes, spleen and lungs were mechanically dissociated and macerated through the 70-micron cell strainer. For lungs, single cell suspension was prepared using enzymatic digestion (Collagenase/DNase) at room temperature for 1 h. Single-cell suspensions were stained with a fixable viability dye, then pretreated with unlabeled anti-CD16/CD32 (Fc Block BD Pharmingen). Cells were then stained in FACS buffer (PBS containing 2.5% fetal calf serum and 0.1% sodium azide) with mixtures of directly conjugated antibodies
Instrument	BD LSR Fortessa was used to collect data for analysis.
Software	All flow data was collected using FACS DIVA (BD Pharmingen) and analyzed using FlowJo V10 Software
Cell population abundance	For adoptive transfer experiments cells were MACS sorted and purity was >95 %.
Gating strategy	FSC-A/SSC-A was used to gate on lymphocytes. The doublets were excluded through FSC-H/FSC-A then SSC-H/SSC-A. Then ,dead cells positive for Live/Dead Blue were excluded. CD8 cells were gated as CD8-apha positive. Donor cells were gated on their distinct congenic marker (s) using CD45/1/CD45.2 gating. A representative gating strategy for different figures is shown in the supplementary file

☒ Tick this box to confirm that a figure exemplifying the gating strategy is provided in the Supplementary Information.
